# Surgical Explantation for Embolized Transcatheter Aortic Valve Replacement Valve at Distal Aortic Arch

**DOI:** 10.1016/j.atssr.2024.11.006

**Published:** 2024-11-23

**Authors:** Andrew Marthy, Junyi Liu, Eduardo Danduch, Saeed Tarabichi, Li Zhang, Sanjay Samy, Chikashi Nakai

**Affiliations:** 1 Department of Cardiothoracic Surgery, Albany Medical Center, Albany, New York

## Abstract

The patient was a 65-year-old man with a history of symptomatic aortic stenosis for which he underwent transcatheter aortic valve replacement (TAVR) with a 26-mm balloon-expandable valve through right femoral artery access. The TAVR valve embolized in the distal transverse arch. An additional 29-mm balloon-expandable valve was deployed at the aortic annulus successfully, and the embolized valve remained in the distal arch. The patient subsequently underwent surgical explantation of the embolized valve with central cannulation and deep hypothermic circulatory arrest. The transverse aortic arch was opened, and the embolized valve was removed. His postoperative course was uncomplicated. He was discharged home on postoperative day 6.

Transcatheter aortic valve replacement (TAVR) valve embolization is rare but can result in critical complications. With the increasing use of TAVR in lower-risk and younger patients, procedural valve embolization will likely become more frequent. However, there are few reports of TAVR complications and even fewer reports of surgical explantation of embolized TAVR valves. Cannulation strategy remains a critical aspect of the safe conduct of cardiopulmonary bypass, to minimize morbidity and to provide a bloodless field in which to operate.^1^ In the case of an embolized valve, an alternative cannulation strategy would be dictated by the location of the embolized valve. Embolized TAVR valves to the aortic arch should be removed because of the risk of embolic stroke and the risk of aortic dissection.^2^^,^^3^ This case report describes a patient whose TAVR valve was embolized at the distal aortic arch and eventually required surgical removal.

The patient was a 65-year-old man with a history of symptomatic aortic stenosis for which he underwent TAVR with a 26-mm balloon-expandable valve through right femoral artery access. When the valve was deployed at the aortic annulus, it embolized into the ascending aorta in response to a premature ventricular contraction (Figure 1A). After additional attempts to remove the valve, it eventually embolized to the distal aortic arch (Figure 1B). Aortography revealed that the valve was embolized between the left common carotid artery (LCA) and the left subclavian artery (LSA), with confirmation that these 2 vessels were patent (Figure 1B). An additional 29-mm balloon-expandable valve was deployed at the aortic annulus successfully. The decision was made to leave the embolized valve in the aortic arch and to follow up the patient on an outpatient basis. Computed tomographic angiography on postprocedural day 0 confirmed that the valve was located at the aortic arch near the origin of the LSA (Figure 2). He was discharged home without symptoms and came back to the cardiac surgery clinic to discuss surgical management 10 days later. Given his potential risk of embolization to the LCA or LSA, the surgical team opted for open surgical explantation of the embolized valve. He underwent full sternotomy, and cardiopulmonary bypass was established with central ascending aorta cannulation to start cooling. Peripheral cannulation through the femoral artery was avoided because of the risk of closure of the valve secondary to retrograde flow from femoral cannulation. With the use of deep hypothermic circulatory arrest, the transverse arch was opened, and the embolized valve was removed from the distal aortic arch. The postoperative course was uncomplicated. He was discharged home on postoperative day 6.

**Figure 1:**
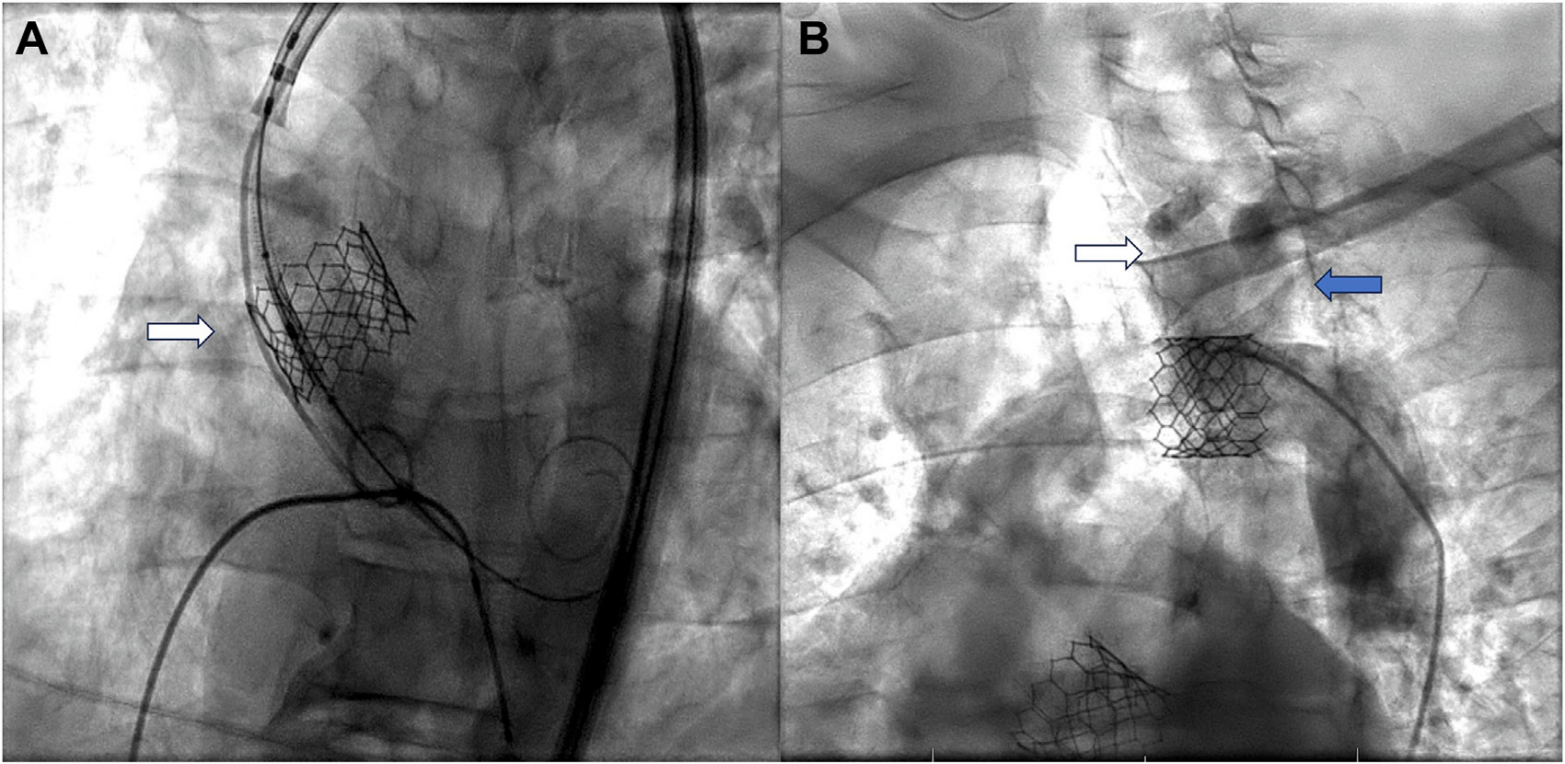
(A) Intraprocedural aortography. Transcatheter aortic valve replacement valve embolized into the ascending aorta as a result of a premature ventricular contraction (white arrow). (B) The transcatheter aortic valve replacement valve lodged in the distal aortic arch between the left common carotid artery and the left subclavian artery after an attempt at pulling back. Patent left common carotid artery (white arrow) and left subclavian artery (blue arrow).

**Figure 2:**
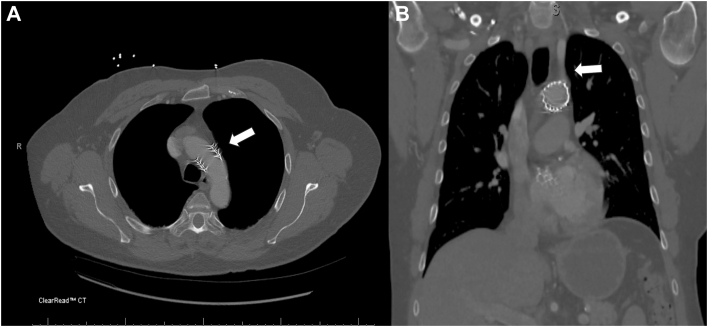
(A) Postprocedural computed tomography. The embolized valve was located at the aortic arch near the origin of the left subclavian artery (white arrow). (B): Patent left subclavian artery (white arrow).

## Comment

The patient presented with an embolized TAVR valve at the distal aortic arch and subsequently required surgical explantation with circulatory arrest. This case demonstrates 2 important aspects: timing for surgical intervention after TAVR valve embolization and cannulation strategy to establish cardiopulmonary bypass.

The timing of surgical intervention after TAVR valve embolization depends on several factors, including the patient’s hemodynamic stability, the location of the embolized valve, and the potential risks associated with leaving the valve in place.^4^ Immediate surgical intervention is necessary for patients with hemodynamic instability or acute complications, whereas patients in stable condition benefit from a more measured approach.^5^ This latter approach allows for comprehensive risk assessment and careful surgical planning, which can lead to improved outcomes. In this case, the patient’s stable condition and absence of life-threatening symptoms allowed for delayed surgery, thus enabling the preparation of an optimal surgical strategy and ultimately resulting in an uncomplicated recovery. For symptomatic patients, such as those experiencing transient ischemic attacks or with evidence of compromised blood flow to major arteries (eg, innominate artery, LCA, LSA), immediate surgical intervention is required to prevent complications such as stroke or limb ischemia. Imaging modalities such as computed tomography, aortography, and carotid artery ultrasound imaging are essential to confirm vascular complications and guide the urgency of surgery. In asymptomatic patients, surgical timing is more flexible and can be elective, on the basis of the potential for future vascular compromise. In this case, the embolized valve in the distal aortic arch posed a risk of future LCA or LSA occlusion. This approach underscores the need for individualized surgical timing, balancing the patient’s current stability with the risks of future adverse events.

**Figure 3:**
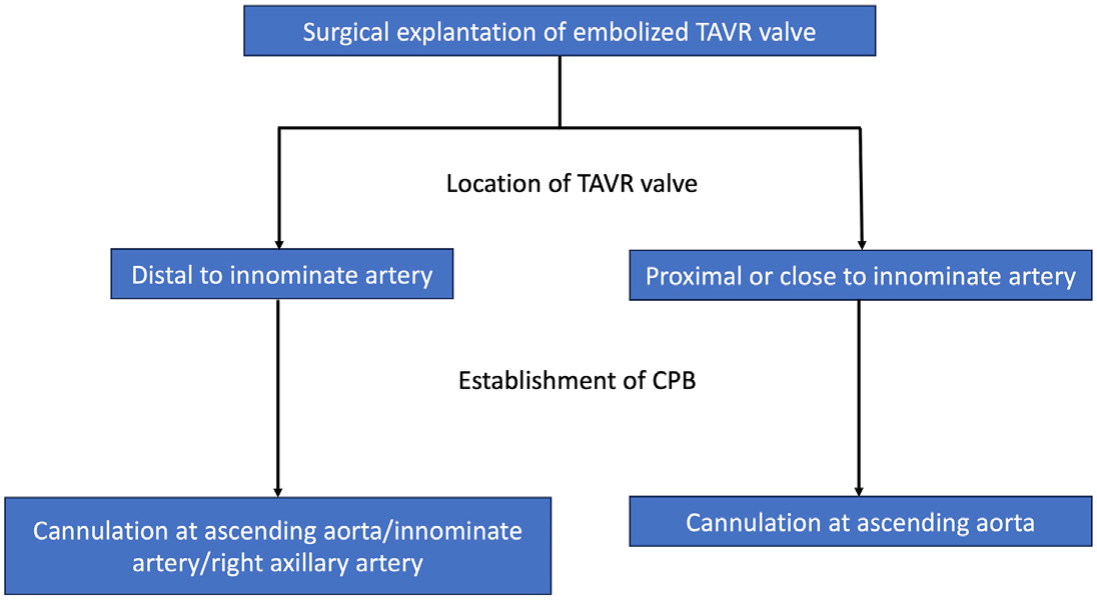
Cannulation strategy to establish cardiopulmonary bypass (CPB) in surgical explantation of an embolized transcatheter aortic valve replacement (TAVR) valve. According to the location of embolized valve, the selection of cannulation site must be considered.

The choice of cannulation strategy in cases of TAVR valve embolization requiring surgical explantation is critical. Patients who undergo TAVR are typically at increased surgical risk, potentially as a result of previous sternotomy or a calcified ascending aorta. In these cases, a peripheral cannulation strategy may be preferred, with minimal aortic manipulation. However, in this patient there was concern that if a peripheral cannulation strategy was used, retrograde flow would result in closing of the embolized TAVR valve and precluding cardiopulmonary bypass flow.^6^ Central ascending aortic or right axillary or innominate artery cannulation allowed for safe initiation of cardiopulmonary bypass (Figure 3). In the case of TAVR valve embolization at the proximal innominate artery or close to the innominate artery, central ascending aortic cannulation would be required to establish cardiopulmonary bypass (Figure 3).

As expected, there is a dearth of literature regarding the optimal management of embolized TAVR valves in the aorta. However, in an era of increasing TAVR use in moderate- to low-risk cases, there needs to be a comprehensive strategy for the heart team and cardiac surgeons to manage these complications, with a focus on “rescuing” the patient from severe morbidity and mortality. The use of central aortic cannulation is supported in cases where peripheral access is compromised or poses a high risk of further embolization. In this patient, the chosen strategy proved effective, thereby enabling a controlled environment for deep hypothermic circulatory arrest and safe removal of the embolized valve from the distal aortic arch.

This case illustrates the importance of individualized cannulation strategies in certain TAVR complications, where standard approaches may not be feasible because of anatomic obstructions or risks associated with the embolized device. The successful outcome further reinforces the efficacy of central cannulation in similar cases.
